# Whole-Genome Sequence of *Sediminibacterium* sp. Strain TEGAF015, Isolated from a Shallow Eutrophic Freshwater Lake in Japan

**DOI:** 10.1128/mra.00882-22

**Published:** 2022-10-17

**Authors:** Yusuke Ogata, Keiji Watanabe, Shusuke Takemine, Chie Shindo, Rina Kurokawa, Wataru Suda

**Affiliations:** a RIKEN Center for Integrative Medical Sciences, Yokohama, Kanagawa, Japan; b Center for Environmental Science in Saitama, Kazo, Saitama, Japan; University of Delaware

## Abstract

The genus *Sediminibacterium* comprises bacteria representing the ubiquitous taxa of freshwater bacterioplankton. Here, we report the whole-genome sequence of *Sediminibacterium* sp. strain TEGAF015, isolated from a shallow eutrophic freshwater lake in Japan.

## ANNOUNCEMENT

The genus *Sediminibacterium* has a worldwide distribution in freshwater rivers, ponds, and lakes (1, 2). To data, only a single species of *Sediminibacterium* has been isolated from the water column rather than freshwater sediments (3). Thus, access to the genomic information of *Sediminibacterium* isolated from water samples might help gain insight into the physiochemical and ecological processes of the respective members belonging to the freshwater taxa.

Here, we report the whole-genome sequence of a *Sediminibacterium* sp. strain, TEGAF015 (JCM 16661), isolated from a sample collected from the surface waters of Lake Teganuma (shallow eutrophic lake), Kashiwa, Chiba, Japan (35°51′40.13″N, 140°01′29.55″E), on 13 September 2008. The sample was filtered through a disposable syringe equipped with a 0.7-μm particle retention glass-fiber filter (Puradisc 25 GF/F disposable filter device; Whatman, Springfield Mill, UK). After filtration, the filtrate was spread on modified Reasoner’s 2A (MR2A) agar plates and incubated at 25°C for 2 to 3 days (4). A single bacterial colony was inoculated into sterile MR2A liquid medium (pH 7.2) and incubated at 25°C for 2 days with reciprocal shaking (120 rpm), and the pure strain cell suspension was preserved as stocks in MR2A broth supplemented with 20% (wt/vol) glycerol at −80°C. Cells from the glycerol stock were inoculated and cultured in MR2A liquid medium, harvested by centrifugation, and used for genomic DNA extraction.

Genomic DNA was extracted from strain TEGAF015 using enzymatic lysis and phenol-chloroform-isoamyl alcohol as previously described (5). Whole-genome sequencing was performed using a Sequel II system (Pacific Biosciences of California, Inc. [PacBio], Menlo Park, CA, USA). The library was prepared with SMRTbell express template preparation kit 2.0 (PacBio) with DNA shearing by gTUBE (Covaris, LLC, Woburn, MA, USA) and with 10- to 15-kb target length. The PacBio reads were converted to circular consensus sequencing (CCS) reads using the ccs software (https://github.com/PacificBiosciences/ccs; v.6.2.0) and assembled using the assembler Canu (v.2.1.1) with specified parameters (minReadLength = 2,200; minOverlapLength = 2,200) (6), and the generated contig was checked for circularization to remap the CCS reads by Minimap2 (v. 2.24-r1122) (7). The obtained genomic sequence was annotated and rotated to start at DnaA using DFAST (https://dfast.nig.ac.jp) (8). Default parameters were used for all software analysis, unless otherwise specified. The obtained reads and generated genomic sequences are summarized in Table 1. The average nucleotide identity by orthology (OrthoANI) and average amino acid identity (AAI) between strains TEGAF015 and Sediminibacterium salmoneum NJ-44^T^ (NBRC 103935) were 84.40% and 89.98%, respectively. Thus, strain TEGAF015 belongs to the genus *Sediminibacterium*.

**TABLE 1:** Summarized data of reads and contigs obtained for *Sediminibacterium* sp. strain TEGAF015

Parameter	Data for strain TEGAF015
No. of quality-passed Sequel reads	30,000
Total bases of quality-passed Sequel reads	421,546,248
*N*_50_ of quality-passed Sequel reads	13,986
Total no. of contigs	1
BioProject accession no.	PRJDB14132
BioSample accession no.	SAMD00520030
Sequence Read Archive (SRA) accession no.	DRR398018
Genome size (bp)	3,055,665
GC content (%)	38.5
GenBank/ENA/DDBJ accession no.	AP026683

Based on annotation results of the TEGAF015 strain genome sequence, a gene predicted to encode polyphosphate kinase (*ppk*) associated with intracellular accumulation of polyphosphate was identified, in addition to putative alanine-, glycine-, and glutamate-dehydrogenase-like protein-encoding genes associated with ammonification. However, a rhodopsin-like protein-encoding gene was absent in the analyzed genomic sequence.

### Data availability.

The chromosome sequence and reads of the strain TEGAF015, the details of which are summarized in Table 1, were deposited in the GenBank/ENA/DDBJ database under accession number AP026683. The BioProject accession number is PRJDB14132. The BioSample accession number is SAMD00520030. The Sequence Read Archive accession number is DRR398018.
